# Clinical Progression: Insights from Longitudinal Studies

**DOI:** 10.1002/alz70857_099678

**Published:** 2025-12-24

**Authors:** Gil D. Rabinovici, Renaud La Joie

**Affiliations:** ^1^ Memory and Aging Center, Weill Institute for Neurosciences, University of California, San Francisco (UCSF), San Francisco, CA, USA; ^2^ Department of Neurology, University of California, San Francisco, San Francisco, CA, USA

## Abstract

This session will examine the clinical progression of atypical Alzheimer's disease (AD) variants, which present unique challenges due to their heterogeneity and earlier onset compared to typical AD. Initial insights into cognitive decline, neuropsychiatric symptoms, protein spread and neurodegeneration trajectories have come from unicentric cohorts, which, despite limited sample sizes, have provided valuable data on the natural history of atypical variants such as Posterior Cortical Atrophy (PCA) and logopenic Primary Progressive Aphasia (lvPPA). Building on these foundational studies, larger multi‐center initiatives, such as the LEADS study, aim to characterize these trajectories more comprehensively, offering critical insights for syndrome‐specific clinical trials.

A key focus of the session will be the ongoing efforts to harmonize clinical and neuroimaging datasets across centers. This harmonization is essential for creating large, syndrome‐specific cohorts, enabling robust analyses of progression and treatment responses in atypical AD. The session will highlight the advantages of standardizing protocols for cognitive assessments, neuroimaging measures, and biomarker collection to ensure consistency and comparability across sites.

By integrating data from diverse cohorts and multi‐center initiatives, these efforts aim to overcome the challenges posed by the rarity and variability of atypical AD. Such approaches not only enhance statistical power but also lay the groundwork for developing innovative therapeutic strategies tailored to the unique needs of individuals with atypical variants, ultimately improving clinical outcomes and advancing the field.

